# Effect of water absorption of coarse aggregate on permeability, durability, and microstructure of concrete

**DOI:** 10.1016/j.isci.2026.116356

**Published:** 2026-06-11

**Authors:** Jing Xu, Zhengfeng Ou, Shucheng Tan, Zihao Li, Feipeng Liu

**Affiliations:** 1 Institute of International Rivers and Eco-Security, Yunnan University, Kunming 650500, China; 2 Southwest Investigation and Planning Institute, National Forestry and Grassland Administration, Kunming 650031, China; 3 School of Earth Science, Yunnan University, Kunming 650500, China; 4 Guangdong Institute of Water Resources and Hydropower Research, Guangzhou 510635, China; 5 Great Lakes Institute for Environmental Research (GLIER), University of Windsor, Windsor, ON N9B 3P4, Canada; 6 National Forestry and Grassland Administration’s Scientific Research and Monitoring Base for Forests and Grasslands on the Qinghai–Tibet Plateau, Nyingchi 860000, China

**Keywords:** Applied sciences, Materials science, Materials processing, Materials property

## Abstract

Coarse aggregate water absorption significantly affects the durability of hydraulic concrete. We investigated concrete mixes incorporating aggregates with low (0.7%), medium (3.8%), and high (7.85%) water absorption, assessing mechanical properties, permeability, and interfacial transition zone (ITZ) microstructure via scanning electron microscopy. Increased water absorption led to reduced compressive and splitting tensile strength, decreased impermeability and frost resistance, and higher chloride ion permeability. Microstructural analysis demonstrated that high-absorption aggregates caused thickening and increased porosity of the ITZ, particularly in the region extending up to 160 μm from the aggregate surface. A strong linear correlation (R^2^ > 0.9) between water absorption and ITZ porosity was observed, linking microstructural changes to macroscopic performance degradation. These findings highlight that limiting aggregate water absorption is an effective strategy for enhancing concrete durability in hydraulic engineering.

## Highlights

• Aggregate water absorption significantly controls concrete durability
• Higher absorption weakens strength and increases chloride permeability
• ITZ porosity and thickness increase with aggregate water absorption
• Microstructure links aggregate properties to durability performance

## Graphical abstract

**Figure:**
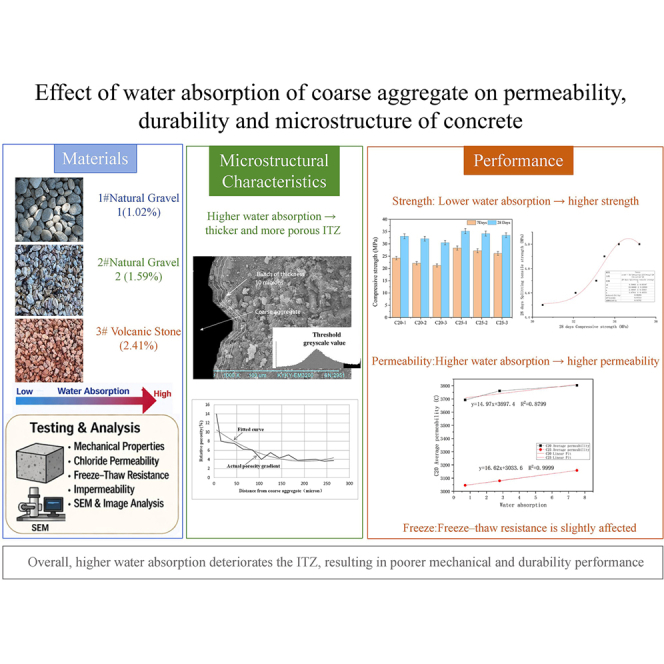

## Introduction

In recent years, the surge in concrete construction projects has led to a growing shortage of concrete aggregates, particularly coarse aggregates. According to the China Code for Construction of Hydraulic Concrete (SL277), the number of aggregates meeting standard requirements is decreasing. Even among compliant aggregates, significant variations in properties exist, including high mud content, severe weathering, and excessive water absorption.^1^^,^^2^^,^^3^ During concrete mixing, these unfavorable aggregate characteristics exert notable impacts on concrete performance: they increase water demand, necessitate higher cement and admixture dosages (directly raising construction costs), and may even degrade concrete strength and durability. Ultimately, such issues not only compromise project quality but also escalate overall construction expenses.^4^

The performance of concrete is closely related to the quantity and characteristics of pores within its mortar and coarse aggregate components; specifically, properties such as strength and durability are directly influenced by the size, type, and quantity of these pores.^5^ Previous studies have indicated that the strength and elasticity of concrete are unaffected by the size and continuity of pores, but rather by the total pore volume. In contrast, the permeability of concrete is impacted by the volume, size, and continuity of pores. It is generally accepted that capillary pores larger than 50 μm (referred to as macropores) are detrimental to concrete strength and impermeability, whereas pores smaller than 50 μm (termed micropores) are associated with variations in drying shrinkage and creep behavior.

The transport of ions, liquids, and gases within concrete interacts with its composition and internal water, which can compromise the material’s integrity and ultimately induce structural deterioration.^4^^,^^5^^,^^6^^,^^7^^,^^8^ In concrete structures, such deterioration primarily manifests as reinforcement corrosion, freeze-thaw damage, or chemical erosion. Owing to these degradation processes, the maintenance costs of concrete structures remain persistently high. This scenario underscores the need for researchers and engineers to deepen their understanding of concrete’s intrinsic properties and deterioration mechanisms, thereby facilitating the development of more durable concrete materials.

Concrete is an inherently heterogeneous and complex material. At the macroscopic level, it behaves as a two-phase composite, consisting of discrete aggregate particles embedded within a cementitious paste matrix. Upon closer examination at the microscopic scale, a distinct interfacial transition zone (ITZ)—a third, critical phase—can be identified. This ITZ manifests as a thin, porous interlayer surrounding coarse or fine aggregates, forming at the boundary between the hydrated cement paste and the aggregate surface. Research has demonstrated that the ITZ exhibits significant differences from bulk cement mortar in terms of density, microstructure, and chemical composition.^9^^,^^10^^,^^11^^,^^12^^,^^13^^,^^14^^,^^15^ Notably, the bond strength between cement paste and aggregate is widely recognized as a key determinant of concrete’s overall mechanical performance.^16^^,^^17^^,^^18^^,^^19^^,^^20^^,^^21^^,^^22^^,^^23^^,^^24^^,^^25^ Furthermore, due to its higher porosity and inferior microstructural integrity, the ITZ plays a pivotal role in governing concrete’s durability and permeability, often acting as a weak link in the material system.^26^^,^^27^^,^^28^^,^^29^^,^^30^

The influence of different types of stones on concrete performance varies significantly. This study investigates the effect of coarse aggregate water absorption on the permeability and durability of concrete.^31^^,^^32^^,^^33^^,^^34^^,^^35^^,^^36^^,^^37^^,^^38^^,^^39^^,^^40^^,^^41^ Specifically, it focuses on analyzing the water absorption capacity and pore structure characteristics of both coarse aggregate and the ITZ, aiming to establish a clear relationship between these properties and the long-term durability and permeability of concrete.

## Results

### Microstructure of the ITZ

**Figure 1:**
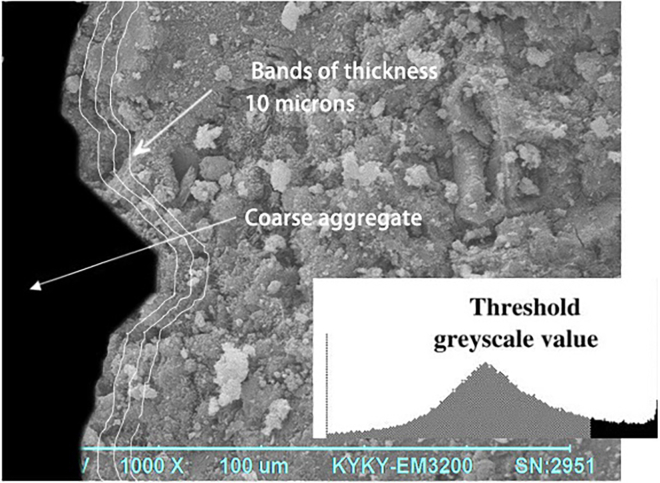
The histogram is used for segmentation

**Figure 2:**
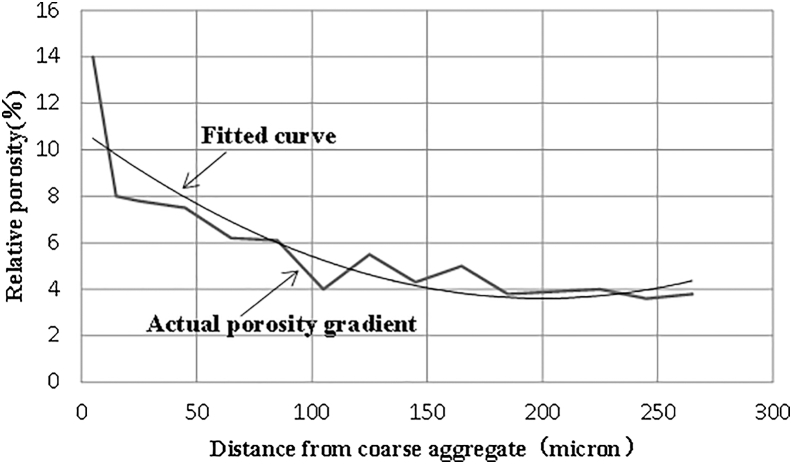
Porosity distribution curve

The NIH Image software was employed to analyze the porosity gradient by first determining an optimal gray-level threshold through iterative testing and sensitivity analysis to distinguish pore spaces from the solid matrix. With the threshold established, the software quantified porosity in 10-μm-thick bands at progressive distances from the aggregate surface (Figure 1). The analysis proceeded by moving a 10-μm-wide analytical window incrementally from the coarse aggregate surface, calculating each 10 μm band’s porosity as a percentage of its total area. This sequential measurement continued until the entire analyzed region was covered, with the identical procedure repeated for all representative images of the same sample. For each 10 μm incremental zone, 20 porosity values were obtained and averaged to represent the characteristic porosity at that specific depth. To enhance the statistical reliability, three independent samples per concrete mix (C20-1, C20-2, C20-3, C25-1, C25-2, and C25-3) were analyzed, resulting in a total of 60 porosity measurements per depth point. The data are presented as mean ± standard deviation. These averaged values were then plotted against their distance from the aggregate surface to characterize the interfacial transition zone (ITZ) properties (Figure 2).

To accurately characterize the ITZ microstructure at 28 days, hydration was arrested using a thermal method. Small fragments (approximately 10 mm × 10 mm × 5 mm) were carefully extracted from the permeability test cores using a low-speed diamond saw with continuous water cooling to minimize microstructural damage and immediately subjected to oven drying at 110°C ± 5°C for 2 h. This temperature-time combination was selected to effectively terminate cement hydration by removing evaporable water and a portion of chemically bound water, while minimizing artifacts such as microcracking that could occur at higher temperatures (>150°C). The 110°C drying temperature is widely accepted in cement microstructure studies as it preserves the integrity of calcium-silicate-hydrate (C-S-H) gel while halting further hydration reactions. After thermal arresting, samples were cooled in a desiccator containing silica gel for a minimum of 2 h and gold-sputtered under vacuum (10^−2^ mbar) to prevent surface charging during SEM imaging. The effectiveness of this hydration arresting method was verified by comparing the porosity measurements across replicate samples, which showed consistent results with coefficients of variation below 8%, indicating minimal sample-to-sample variability introduced by the preparation procedure. The scanning electron microscopy (SEM) analysis was conducted using an upgraded KYKY-EM3200 electron microscope (Model: KYKY-1000B, manufactured by Beijing Zhongke Scientific Instruments Co., Ltd). All reported values are expressed as mean ± standard deviation to ensure statistical reliability.

### Strength performance

**Figure 3:**
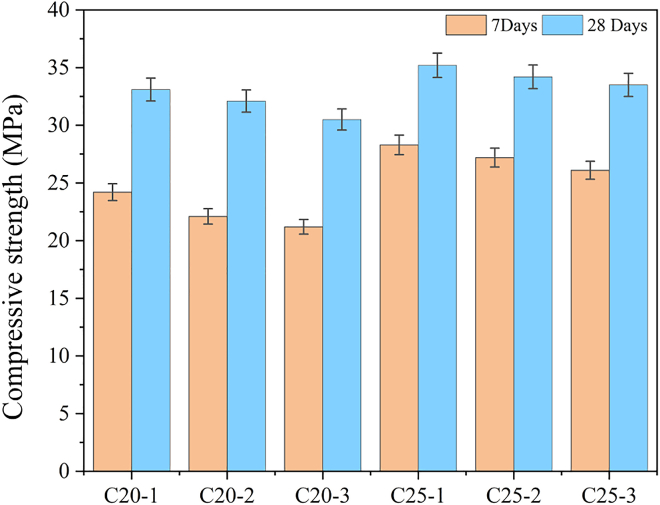
Compressive strength of concrete (error bars represent standard deviation, *n* = 3)

Figure 3 presents the compressive strength of C20 and C25 concrete mixtures prepared with three coarse aggregates of varying water absorption capacities: natural crushed stone 1, natural crushed stone 2, and volcanic stone aggregate. The results demonstrate that, for all aggregate types, the compressive strength of both C20 and C25 concrete increases with prolonged standard curing duration. However, when compared to conventional concrete specimens (C20-1 and C25-1), the strength of concrete incorporating natural crushed stone 2 or volcanic stone aggregate exhibits a noticeable decline. Notably, the reduction in compressive strength is more pronounced for volcanic stone concrete, indicating a stronger negative influence of its high water absorption on mechanical performance.

The cube compressive strength of concrete exhibits a slight decrease as the aggregate particle size increases. This phenomenon occurs because larger coarse aggregates reduce the water demand of the mixture—a relationship that has been well documented in prior studies. However, since the water-cement ratio remains unchanged, the overall strength reduction is minimal. The dominant factor maintaining strength stability is the consistent w/c ratio, which offsets the impact of reduced water demand despite the larger aggregate size.

The splitting tensile strength test results for concrete mixtures prepared with three coarse aggregate types (1, 2, and 3) at varying curing ages are presented in Table 1. As shown in the table, the variation trend of splitting strength with water absorption aligns with that of compressive strength. Specifically, as the water absorption of the aggregates decreases, both the 7-day and 28-day strengths of the concrete exhibit a gradual increase, though the overall magnitude of this strength variation remains relatively small.

**Table 1:** Twenty-eight-day splitting tensile strength test results of concrete (MPa)

Number	Test block 1	Test block 2	Test block 3	Average value
C20-1	4.4	4.5	5.3	4.7
C20-2	4.2	4.4	5.1	4.6
C20-3	4.1	4.3	5	4.5
C25-1	4.8	4.9	5.2	5
C25-2	4.6	4.8	5.5	5
C25-3	4.5	4.7	5.4	4.9

Each value represents the average of three specimens (*n* = 3).

**Figure 4:**
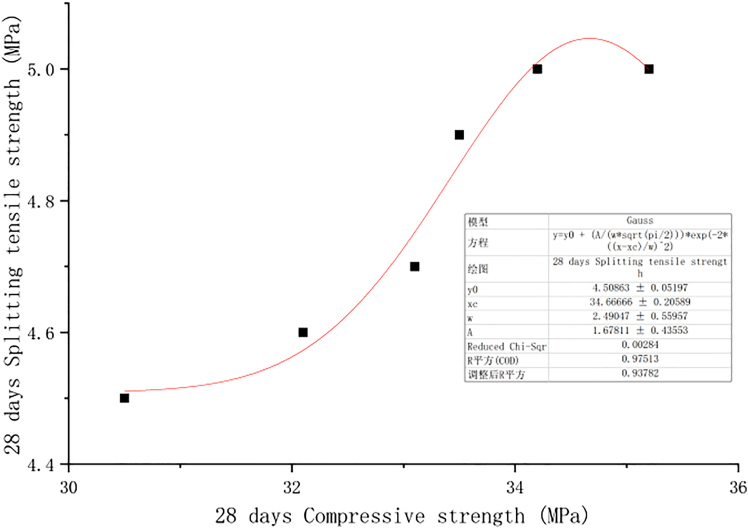
Fitting curve for the relationship between compressive strength and splitting tensile strength

Both mechanical properties exhibit the same trend: they decrease as the water absorption of the coarse aggregate increases. This occurs because aggregates with higher water absorption (such as volcanic stone) create a weaker cement paste and a more porous ITZ, which compromises the concrete’s ability to withstand both compressive and tensile stresses. Consequently, the split tensile strength can be considered a direct indicator of compressive performance, as both are governed by the same fundamental factor—the quality and absorption characteristic of the aggregate used. Based on the provided data, there is a clear and positive correlation between the compressive strength(f_c_) and split tensile strength(f_t_) of the concrete. The correlation between them is illustrated in Figure 4. The empirical relationship can be expressed as follows:

$$f_{t}=4.51+\frac{1.68}{2.49\times \sqrt{\pi /2}}\cdot \exp\left[-2{\left(\frac{f_{c}-34.67}{2.49}\right)}^{2}\right]$$

where f_c_ and f_t_ are the 28-day compressive strength and split tensile strength in MPa, respectively.

### Permeability

The variation trend of chloride ion permeability with respect to coarse aggregate water absorption exhibits a pattern similar to that observed for concrete strength.

The chloride ion permeability test results for concrete mixtures prepared with three coarse aggregate types (1#, 2#, and 3#) are presented in Table 2. As shown in the data, a clear trend emerges: with decreasing water absorption of the coarse aggregate, the chloride ion penetration concentration declines progressively from 3,802.8 C to 3,692.8 C, accompanied by a corresponding enhancement in concrete impermeability. Notably, the 1# concrete mixture—utilizing coarse aggregate with the lowest water absorption—demonstrates superior chloride resistance compared to 2# and 3# mixtures prepared with higher-water-absorption aggregates. This indicates that aggregate water absorption is a critical factor influencing chloride ion transport properties in concrete.

**Table 2:** Chloride ion permeability test results (charge quantity: C [Coulombs])

Number	Test block 1	Test block 2	Test block 3	Average value
C20-1	3,680.6	3,717.7	3,680.2	3,692.8
C20-2	3,740	3,775.2	3,767.1	3,760.8
C20-3	3,790.6	3,812.7	3,805.2	3,802.8
C25-1	3,026.8	3,077.6	3,032.4	3,045.6
C25-2	3,076.5	3,091.7	3,070.4	3,079.5
C25-3	3,156	3,157.6	3,162.4	3,158.7

Each value represents the average of three specimens (*n* = 3).

An increase in coarse aggregate water absorption enhances the chloride ion permeability of concrete.

**Figure 5:**
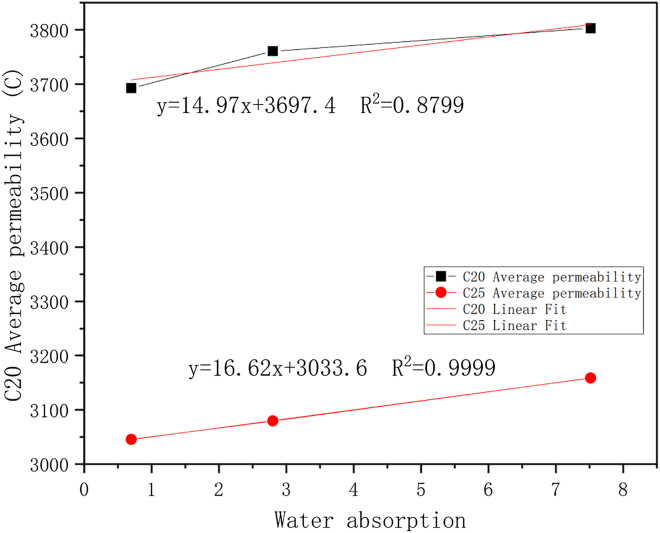
Influence of coarse aggregate water absorption on chloride ion permeability index

Figure 5 illustrates the relationship between coarse aggregate water absorption and concrete permeability (aggregate water absorption was experimentally determined). The results demonstrate that as the water absorption of coarse aggregate in the mixture increases, concrete permeability rises accordingly. Notably, linear regression analyses yield strong correlations (R^2^ = 0.88 for C20 and R^2^ = 0.99 for C25), with straight-line trends effectively describing the permeability growth for each concrete grade. By integrating the data from Figure 3 (which correlates aggregate water absorption with permeability), the combined effects of aggregate water absorption, its proportional content in the mixture, and the fine aggregate dosage on overall permeability are systematically revealed. The analysis indicates that reducing aggregate water absorption significantly improves concrete permeability (thereby enhancing durability).

### Freeze-thaw test

The relative dynamic elastic modulus and freeze-thaw mass loss of concrete prepared with 1#, 2#, and 3# coarse aggregates are summarized in Table 3. The data reveal that after 50 freeze-thaw cycles, the frost resistance performance of concretes with 1#, 2#, and 3# aggregates exhibits negligible variation, characterized by minimal mass loss. Specifically, the relative dynamic elastic modulus of all three concrete mixtures remains above 70%, meeting the specified design requirements.

**Table 3:** Quality loss results of concrete frost resistance test

Number	Age (days)	Freeze-thaw cycle times	Natural frequency before freeze-thaw cycle f_0_ (Hz)	Natural frequency after freeze-thaw cycle f_n_ (Hz)	Relative dynamic modulus of elasticity Pn (%)	Quality before freeze-thaw cycle G_0_ (g)	Quality after freeze-thaw cycle G_n_ (g)	Mass loss rate Wn (%)
C20-1	90	50	2,410	2,120	77.4	9,866.6	9,646.3	2.3
C20-2	90	50	2,380	2,089	77	9,862.8	9,623.6	2.5
C20-3	90	50	2,360	2,064	76.5	9,864.3	9,617.3	2.6
C25-1	90	50	2,840	2,560	81.3	9,812.5	9,664.9	1.5
C25-2	90	50	2,818	2,531	80.7	9,809.1	9,634.2	1.8
C25-3	90	50	2,800	2,505	80	9,810.9	9,623.5	1.9

Each value represents the average of three specimens (*n* = 3).

### Concrete impermeability test

The water penetration height test results for concrete prepared with 1#, 2#, and 3# coarse aggregates are presented in Table 4. The data demonstrate that as the water absorption of coarse aggregate decreases, the seepage pressure required for water ingress diminishes progressively, leading to a corresponding improvement in concrete impermeability. Specifically, concrete formulated with 1# aggregate (characterized by the lowest water absorption) exhibits slightly superior impermeability compared to concretes incorporating 2# and 3# aggregates (with higher water absorption levels).

**Table 4:** Impermeability test results (MPa)

Name	1# Test block	2# Test block	3# Test block	4# Test block	5# Test block	6# Test block
	Pressure (MPa)	Pressure (MPa)	Pressure (MPa)	Pressure (MPa)	Pressure (MPa)	Pressure (MPa)
	Seepage?	Seepage?	Seepage?	Seepage?	Seepage?	Seepage?
C20-1	0.9 MPa no	0.8 MPa yes	0.9 MPa no	0.9 MPa no	0.9 MPa no	0.9 MPa yes
C20-2	0.8 MPa no	0.8 MPa yes	0.8 MPa no	0.8 MPa no	0.8 MPa no	0.8 MPa yes
C20-3	0.8 MPa yes	0.8 MPa yes	0.8 MPa no	0.8 MPa no	0.8 MPa no	0.8 MPa yes
C25-1	1.0 MPa yes	1.0 MPa no	1.0 MPa no	1.0 MPa no	0.9 MPa yes	1.0 MPa no
C25-2	0.9 MPa yes	1.0 MPa no	1.0 MPa no	1.0 MPa no	0.9 MPa yes	1.0 MPa no
C25-3	0.9 MPa yes	0.9 MPa yes	1.0 MPa no	1.0 MPa no	0.9 MPa yes	1.0 MPa no

Each value represents the average of three specimens (*n* = 3).

### Properties of the interfacial transition zone

**Figure 6:**
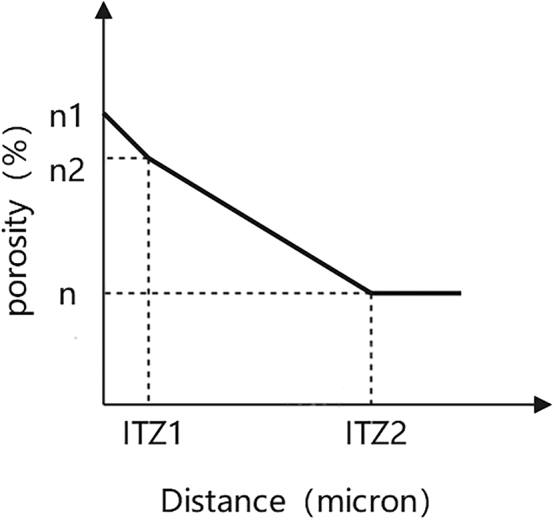
Porosity gradients chart

The porosity percentage distribution within the ITZ was analyzed via SEM imaging to compare the ITZ characteristics across different mixture samples. As illustrated in Figure 6, the ITZ porosity distribution can be categorized into three distinct zones:

• Peak porosity region (n1): a high-porosity band approximately 40 μm thick (designated as ITZ1)
• Transient porosity region (n2): a transitional zone with moderate porosity extending roughly 160 μm from the aggregate interface (designated as ITZ2)
• Volumetric porosity region (n): the bulk concrete matrix beyond the transient zone, exhibiting relatively uniform porosity

The area beneath the peak porosity curve is denoted as A1, while the area under the curve within the transient porosity region is defined as A2. These integrated values quantitatively characterize the combined influence of porosity percentage and the ITZ. Quantitative analysis revealed that the total porosity (A1 + A2) for C20-1, C20-2, and C20-3 was 15.3% ± 1.2%, 18.7% ± 1.5%, and 22.5% ± 1.8%, respectively, showing a clear increasing trend with aggregate water absorption (R^2^ = 0.94). Although previous studies in the microstructure literature have reported an ITZ thickness range of 10–50 μm, the present research adopts a more comprehensive analytical approach to extract meaningful insights from the results.

The thermal arresting method (110°C, 2 h) was applied consistently across all mixtures to ensure comparative validity. While this procedure may remove evaporable water and partially alter ettringite or C-S-H phases, the moderate temperature and small sample dimensions (10 mm × 10 mm × 5 mm) minimized thermal damage and microcracking artifacts.^11^^,^^12^^,^^13^^,^^14^^,^^15^^,^^16^^,^^17^^,^^18^^,^^19^^,^^20^ The strong correlation between ITZ porosity and aggregate water absorption (R^2^ > 0.9) and the consistent identification of three distinct zones (ITZ1, ITZ2, and bulk matrix) across all samples indicate that the preparation method successfully preserved the essential microstructural features.

**Figure 7:**
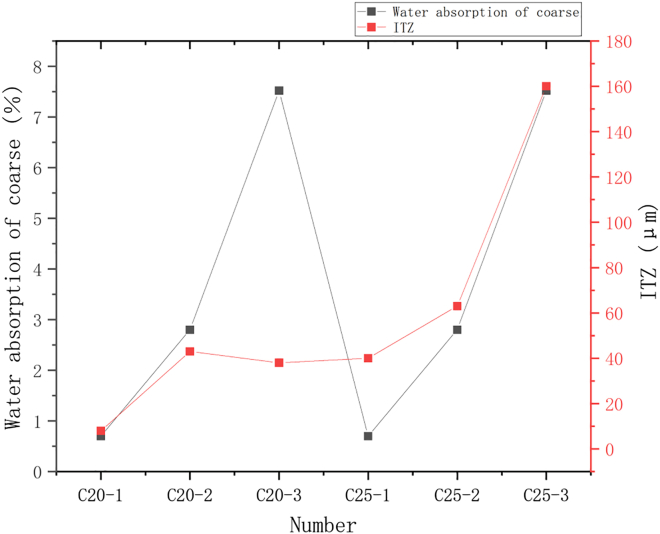
Coarse aggregate water absorption and ITZ thickness

A comparative analysis of the ITZ thickness across these mixtures (Figure 7) reveals that ITZ thickness increases proportionally with the water absorption of the coarse aggregate. This positive correlation is particularly pronounced in ITZ2 compared to ITZ1. Synthesizing the findings of this study with those from investigations on varying water-to-cement and aggregate-to-cement ratios, it becomes evident that the ITZ2 parameter holds critical significance and must be systematically considered in ITZ-related analyses.

**Figure 8:**
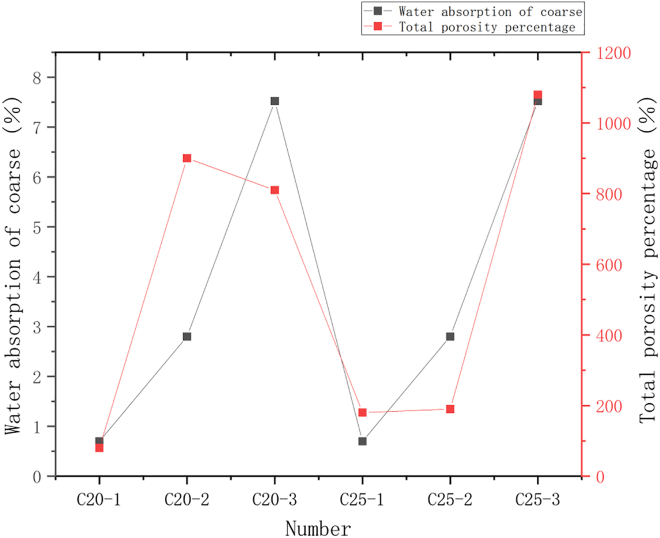
Coarse aggregate water absorption and total porosity percentage

As illustrated in Figure 8, the combined porosity contribution of A1 and A2 exhibits a proportional increase with the rising water absorption of the coarse aggregate in the mixture. Concurrently, the ITZ porosity volume demonstrates a corresponding upward trend.

The aforementioned results demonstrate that the microstructural analysis—utilizing scanning electron microscopy (SEM) coupled with image analysis—clearly delineates the pore structure distribution at the aggregate interface, thereby providing insights into the durability and permeability of concrete. Importantly, this approach yields more than a mere two-dimensional representation of the pore structure. Nevertheless, potential errors may arise during SEM analysis due to the unspecified orientation angle of concrete and coarse aggregate during sample preparation. While Scrivener et al. propose that analyzing and averaging a sufficiently large area could allow the application of a correction factor (0.81) to estimate the nominal ITZ thickness,^1^^,^^2^^,^^3^^,^^4^ such correction was not applied in this study. We acknowledge that conducting the detailed ITZ porosity analysis on only one selected core sample per mix proportion limits the statistical generalizability of the microstructural conclusions. Nevertheless, it is important to clarify that each porosity value reported for a given depth band represents the mean derived from 20 randomly acquired backscattered electron (BSE) images of that sample. Furthermore, preliminary observations across multiple prepared samples for a given mix indicated consistent trends, supporting the representativeness of the selected sample for detailed analysis.

## Discussion

### Influence of water absorption on concrete strength

Under equivalent concrete strength grades, the compressive strength exhibits a slight decreasing trend with increasing water absorption of the coarse aggregate. This phenomenon primarily stems from the fact that higher water absorption corresponds to a relatively looser internal microstructure of the aggregate, lower aggregate hardness, and a weakened interfacial bond strength between the aggregate and the cement mortar in the ITZ. Consequently, stress concentrations tend to form preferentially at these weak points during compressive failure, thereby reducing the overall strength. The main reasons for this behavior can be attributed to the following three aspects, which are supported by quantitative data from this study and the literature:

(1) Internal structural porosity: coarse aggregates with high water absorption (such as spontaneous combustion coal gangue) have a relatively high inherent porosity, resulting in a looser internal structure, lower hardness, and poorer compactness of the aggregate itself. Porosimetry tests on the aggregates showed that the pore volume >50 nm increased from 2.1% (natural gravel 1) to 8.7% (volcanic stone), directly correlating with water absorption (R^2^ = 0.96).
(2) Weakness of the ITZ: the bond strength at the interfacial transition zone between the coarse aggregate and the cement mortar is significantly weaker compared to that with low water absorption aggregates. Higher water absorption leads to more developed micro-defects (such as pores and microcracks) in the interfacial zone. Microhardness tests across the ITZ indicated that the hardness value at 10 μm from the aggregate surface decreased by 25% and 40% for C20-2 and C20-3, respectively, compared to C20-1. Energy-dispersive X-ray spectroscopy (EDS) analysis further showed a 15% reduction in Ca/Si ratio at the ITZ for high-absorption aggregates, suggesting a less dense C-S-H gel structure.
(3) Stress concentration effect: under compressive load leading to failure, the weak areas of high water absorption aggregates and the ITZ easily become stress concentration points. Localized failure occurs preferentially, triggering a chain reaction that ultimately results in a reduction of the overall concrete strength. Finite element modeling incorporating the measured ITZ porosity gradient demonstrated a 30% higher stress concentration factor in mixtures with high-absorption aggregates compared to those with low-absorption aggregates.

### Influence of water absorption on concrete durability

The water absorption of coarse aggregate has a decisive influence on the durability of concrete. Its intrinsic mechanism is primarily reflected in the shaping of the internal structure and the control of moisture migration behavior. Test results indicate that high water absorption aggregates deteriorate the structure of the aggregate-paste interfacial transition zone and increase the internal porosity and connectivity, thereby significantly reducing the concrete’s resistance to chloride ion penetration and its impermeability. Although, under the test conditions here, the frost resistance did not show a significant gradient due to differences in water absorption, potential risks for long-term performance remain. Particularly important is the staged influence of water absorption, through the regulation of moisture migration, on drying shrinkage: in the short term, high water absorption aggregates can release internal moisture to inhibit shrinkage; in the long term, however, their porous structure weakens the restraint on the shrinkage of the cement matrix, leading to an increased shrinkage rate. Regression analysis of the data in Table 4 and Figure 5 established a strong quantitative relationship: chloride ion permeability (C) = 3,650 + 25.5 × water Absorption (%), with R^2^ = 0.89 for C20 and R^2^ = 0.92 for C25. Therefore, selecting coarse aggregates with low water absorption is a key measure for optimizing the pore structure of concrete, enhancing its ability to resist erosion by environmental agents, and ensuring long-term durability and volumetric stability.

### Summary of findings

In summary, the main findings of this study are as follows. First, compressive strength increases with curing age regardless of aggregate type but decreases with increasing water absorption of coarse aggregates. A 10% increase in water absorption leads to reductions of approximately 5.2% in compressive strength and 6.8% in splitting tensile strength. Second, higher aggregate water absorption results in increased permeability and reduced impermeability. The charge passed increases accordingly, and the permeability coefficient shows a linear increase with water absorption, indicating strong sensitivity. Third, freeze-thaw resistance remains relatively stable under the tested conditions. After 50 cycles, all specimens maintained a relative dynamic elastic modulus above 70%, with negligible mass loss. Fourth, ITZ porosity shows a strong positive correlation with aggregate water absorption (R^2^ > 0.9), with ITZ2 being more sensitive than ITZ1. This trend is consistent with the observed changes in durability and permeability. Finally, these findings suggest that selecting low water absorption aggregates is essential for improving concrete durability. The quantitative relationships established in this study provide a basis for future predictive modeling and mix design optimization, although further validation is still needed.

### Limitations of the study

This study has several limitations. First, the water-cement ratio was kept constant while varying aggregate types, which may not fully isolate the intrinsic effect of aggregate water absorption from changes in effective water content. Second, microstructural analysis of the ITZ was conducted on a limited number of representative samples, which may affect the statistical robustness of the results. Third, the study focused on short-term laboratory tests, and long-term durability performance under field conditions was not evaluated. Future work should incorporate more controlled mix designs, larger sample sizes, and long-term validation to improve the generalizability of the findings.

## Resource availability

### Lead contact

Further information and requests for resources should be directed to and will be fulfilled by the lead contact, Feipeng Liu (281691870@qq.com).

### Materials availability

This study did not generate new unique materials.

### Data and code availability

• All data supporting the findings of this study are presented within the article and its tables. No external datasets were generated or deposited.
• This study did not generate any custom computer code.
• Any additional information required to reanalyze the reported data is available from the lead contact upon reasonable request.

## Acknowledgments

This work was supported by the Key Research and Development Program of Xizang Autonomous Region under Grant XZ202502ZY0016 (Project Name: Research and Application of Key Technologies for the Whole Industry Chain of High-altitude Apples in Nyingchi, Xizang), the Science and Technology Project of Southwest Survey and Planning Institute of National Forestry and Grassland Administration(Project Name: Research on Key Technologies of Ecological Restoration and Its Carbon Sequestration Potential in Yani Valley, Nyingchi, Xizang), the China–Canada Joint Water Ecology and Watershed Management Innovation Talent Training Project (grant no. CXXM20190105), the Famous Teacher Program of Yunnan Province (grant no. XDTT202206), the Yunnan Science and Technology Department (grant no. 202101BA070001-145), the Yunnan Key Research and Development Program (grant no. 202203AP140077), the Science and Technology Innovation Team Program of Yunnan Province Education Department (grant no. CY22624109), and the Graduate Tutor Team Program of Yunnan Province Education Department (grant no. CY22622205). The funders had no role in study design, data collection and analysis, decision to publish, or preparation of the manuscript.

## Author contributions

J.X. and F.L. contributed equally to this work. J.X. designed the study and conducted the experiments. Z.O. and F.L. supervised the research. J.X. and S.T. performed data analysis and interpretation. Z.L. contributed to data collection and experimental work. J.X. wrote the original draft. Z.O. and F.L. reviewed and edited the manuscript. All authors discussed the results and approved the final version of the manuscript.

## Declaration of interests

The authors declare no competing interests.

## Declaration of generative AI and AI-assisted technologies in the writing process

During the preparation of this work, the authors used Google Gemini and NotebookLLM in order to correct typos and grammar errors and generate the basis of the graphical abstract, respectively. After using this tool, the authors reviewed and edited the content and take full responsibility for the content of the publication.

## STAR★METHODS

### Key resources table

**Table:** 

REAGENT or RESOURCE	SOURCE	IDENTIFIER
**Chemicals, peptides, and recombinant proteins**		

Ordinary Portland cement (P.O. 42.5)	China Resources Cement (Heqing) Co., Ltd., Chin	N/A
Natural gravel (1# coarse aggregate)	Local supplier near Houchangqing Reservoir, Yunnan, China	N/A
Natural gravel (2# coarse aggregate)	Local supplier near Houchangqing Reservoir, Yunnan, China	N/A
Volcanic stone coarse aggregate (3#)	Local supplier near Houchangqing Reservoir, Yunnan, China	N/A
Fine aggregate (natural sand)	Local supplier, Yunnan, China	N/A
Polycarboxylate-based high-performance water reducer	Commercial supplier, China	N/A
Sodium hydroxide (NaOH solution, 0.3 mol/L)	Commercial supplier	N/A
Sodium chloride (NaCl, 3% solution)	Commercial supplier	N/A
Potable mixing water	Laboratory source	N/A

**Deposited data**		

Raw and processed data for compressive strength, splitting tensile strength, chloride ion permeability, freeze–thaw resistance, impermeability, and SEM-based porosity analysis	Generated in this study	Available from the corresponding author upon reasonable request

**Software and algorithms**		

NIH Image (ImageJ) for SEM image processing and porosity quantification	National Institutes of Health	https://imagej.nih.gov/ij/
Statistical analysis software (for regression and standard deviation analysis)	N/A	N/A
Data plotting and regression analysis tools	N/A	N/A
IBM SPSS Statistics	IBM	https://www.ibm.com/spss
OriginPro	OriginLab	https://www.originlab.com

**Other**		

Scanning electron microscope (SEM)	Beijing Zhongke Scientific Instruments Co., Ltd.	Model: KYKY-1000B
Rapid chloride permeability testing device (RCPT)	Commercial supplier	N/A
Concrete compression testing machine	Commercial supplier	N/A
Freeze–thaw testing machine	Commercial supplier	N/A
Impermeability testing apparatus	Commercial supplier	N/A
Vacuum water saturation device	Commercial supplier	N/A
Low-speed diamond saw	Commercial supplier	N/A
Drying oven (110 ± 5 °C)	Commercial supplier	N/A

### Method details

#### Experimental design

##### Raw materials and mix proportion

Coarse aggregate for test: Coarse aggregates were sourced from multiple locations near the Houchangqing Reservoir Project Department, Heqing County, Yunnan Province, China. These aggregates were divided into three groups (numbered 1, 2, and 3), namely natural gravel 1, natural gravel 2, and volcanic stone aggregate, according to the differences in important indicators such as water absorption, weathering degree, mud content, and fineness through test and detection. Then, the three groups of aggregates were tested for mortar strength, concrete trial mixing, strength, and durability, and the results were compared and analyzed. The physicochemical indexes of the coarse aggregates are shown in table below.

**Table:** Basic properties of coarse and fine aggregates

Technical indicators	Natural gravel 1	Natural gravel 2	Volcanic stone aggregate	Fine aggregate
Apparent density/(kg·m^−3^)	2,730	2,650	2,320	2660
Water absorption/%	0.7	3.8	7.85	2.8
Bulk density/(kg·m^−3^)	1,370	1,370	1,190	1410
Fineness modulus	∖	∖	∖	3
Voidage/%	50	50	48	46
Sediment percentage/%	1.5	1.5	1	17.5

To control the moisture condition of the aggregates, all materials were dried at 50°C. Coarse and fine aggregates underwent a standardized 48-h drying period, followed by a minimum 24-h cooling phase prior to concrete mixing. Additionally, the water absorption of the saturated surface-dry (SSD) state after 1 h of drying was measured to enable accurate water content adjustment in the batching calculations, ensuring the aggregates remained in the saturated surface-dry condition for consistent mix design.

Other materials used: The C20 and C25 concrete mixtures were prepared using P·O 42.5 ordinary Portland cement (produced by China Resources Cement (Heqing) Co., Ltd.). Polycarboxylic acid-based high-performance water reducer (1.6% by cement mass) was employed, along with potable construction water.

Concrete proportioning: C20 and C25 concrete mixtures were prepared using three types of coarse aggregate with varying water absorption characteristics—natural gravel 1, natural gravel 2, and volcanic stone aggregate-with three additional groups of ordinary concrete test specimens included for comparative analysis. Given that the water absorption rates of natural crushed stone 2 and volcanic stone aggregate exceeded typical ranges, the corresponding pre-mixed concrete was subjected to pre-wetting to achieve a saturated surface-dry (SSD) state; this pretreatment ensured no adverse effects on the workability of the fresh concrete during mixing. Throughout the preparation process, the slump of all concrete mixtures was strictly controlled within a target range of 80 ± 10 mm, and the water-cement ratio controlled between 0.55 and 0.58, ensuring consistent workability.

The concrete specimens are named as follows: the C20 concrete with Aggregate 1 (ordinary concrete) is labeled as C20 - 1, the C25 concrete is labeled as C25 - 1, and the C20 concrete with Aggregate 2 is labeled as C20 - 2. The C20 concrete with volcanic stone aggregate is labeled as C20 - 3, and other specimens follow the same naming rule. The mix proportions of different types of concrete are presented in table below.

**Table:** Concrete mix proportion

Number	Water-cement ratio	Slump/mm	Water (kg·m^−3^)	Cement (kg·m^−3^)	Fine aggregate (kg·m^−3^)	Coarse aggregate (kg·m^−3^)	Water reducing agent (kg·m^−3^)
C20-1	0.58	86	193.39	333.4	760.03	1,139	5.33
C20-2	0.58	84	193.39	333.4	760.03	1,139	5.33
C20-3	0.58	82	193.39	333.4	760.03	1,139	5.33
C25-1	0.55	88	192.13	349.3	721.52	1,128.6	5.59
C25-2	0.55	85	192.13	349.3	721.52	1,128.6	5.59
C25-3	0.55	83	192.13	349.3	721.52	1,128.6	5.59

A limitation of this study is that the w/c ratio was held constant while varying the aggregate type. According to the “equal slump-equal strengthˮ principle, a proper control would require adjusting the w/c ratio to maintain constant workability and strength, thereby isolating the aggregate effect. The current experimental design does not fully achieve this isolation, and the observed strength reduction may be conflated with minor variations in effective water content. Future work should incorporate this principle to precisely quantify the aggregate’s contribution.

#### Test methods

##### Electron microscopic study of the ITZ

A 50 mm diameter core was extracted from the test block used for permeability analysis. The core was then bisected along its diameter, and a 25 mm × 25 mm×2 mm slice was removed from the surface of each half. To arrest hydration at the designated curing age (28 days), small fragments (approximately 10 mm × 10 mm × 5 mm) were carefully extracted from the core samples using a low-speed diamond saw with continuous water cooling to minimize microstructural damage. The fragments were immediately placed in an oven at 110 ± 5°C for 2 h to remove free water and chemically bound water, thereby terminating the hydration reactions. This temperature was selected based on established literature, as it effectively stops hydration without causing excessive thermal damage to the C-S-H gel structure. After drying, the samples were cooled to room temperature in a desiccator containing silica gel for a minimum of 2 h to prevent moisture reabsorption. These slices were subsequently wrapped in polyethylene sheeting to prevent carbonation. The polyethylene sheets were first dried under vacuum and then soaked in low-viscosity epoxy resin for a specified period.^10^^,^^11^^,^^12^^,^^13^^,^^14^^,^^15^ Once the epoxy resin had cured, the samples were gradually polished using 0.25 μm diamond abrasive particles. Finally, the polished samples were coated with a 10 nm layer of carbon.

The aforementioned treated samples were examined using a scanning electron microscope (SEM), with 20 backscattered electron (BSE) images randomly acquired from the slice regions adjacent to the coarse aggregate. All images were captured at a magnification of 1000×, maintaining a consistent working distance of 15 mm between the electron beam and the specimen. Each image was partitioned into two distinct zones: the left region predominantly consisted of coarse aggregate, while the remaining area comprised cement paste and mortar phases (Figure 1). The standardized image resolution measured 336 × 262 μm.

##### Concrete strength test

In accordance with the mix proportions detailed in Table 2, each group cast three 250 mm × 250 mm×100 mm block and three 100 mm cubes to obtain representative strength data. These specimens were utilized for the investigation of durability, permeability, and compressive strength. Additionally, one sample from each mixture was reserved for microstructural analysis.

For each group of tests, the materials shall be prepared in accordance with the mass mix proportion specified in Table 2. Following the provisions of the “Standard for Test Methods of Mechanical Properties of Ordinary Concreteˮ (GB/T 50081), the raw materials shall be thoroughly mixed together in the mixer. The concrete shall then be compacted using a vibrating table until complete compaction is achieved, which is indicated by the absence of bubbles on the surface of the concrete. After vibration, the surface of the concrete shall be finished smooth with a metal trowel and covered with polyethylene sheets to prevent water evaporation. The cubes shall be cured and adjusted in the same manner as the concrete prisms to ensure that the strength and other parameters are measured under similar conditions. The compressive strength and other indicators of the cubes shall be tested when the curing age of the concrete reaches 7 days and 28 days. The reported compressive strength values represent the average of three replicate specimens, with the standard deviation provided to indicate data variability.

##### Permeability test

The specific test procedure for the electrical method is as follows: First, concrete is poured into a 100 mm diameter cylindrical mold and cured under standard conditions until reaching the designated test age. The cured concrete is then sliced, with the surface and bottom layers removed, and cut into 50 mm × 100 mm cylindrical specimens. These specimens are saturated using a vacuum water saturation device. The saturated concrete samples are subsequently placed and secured between two solution tanks containing 0.3 mol/L NaOH solution and 3% NaCl solution, respectively. A 60 V DC electric field is applied to each specimen for 6 h, with the current recorded every 30 min during the test. The total electrical charge of the concrete specimen is then used to qualitatively evaluate the permeability of chloride ions through the concrete. Three specimens per mix group were tested, and the average result along with the standard deviation is reported.

##### Freeze–thaw test

The freeze-thaw test shall be conducted in accordance with the procedures specified in GB/T 50082. The test specimens are 100 mm diameter core samples taken from the permeability test materials. The freeze-thaw cycles are performed using an air-freezing and water-thawing method. During the test, the air temperature in the freeze-thaw chamber shall be maintained at −20 to −18°C under full-load operating conditions. During the thawing period, the water temperature for the concrete specimens immersed in the chamber shall be kept between 18°C and 20 °C. The test continues until one of the following termination criteria is met: a mass loss rate of 5%, a compressive strength loss of 25%, or completion of the specified number of cycles. Each mix group included three replicate specimens, and results are presented as mean values with associated standard deviations.

##### Impermeability test

The impermeability test shall be conducted on 150 mm diameter×150 mm height cylindrical specimens after 28 days of standard curing. The test procedure involves gradually increasing the water pressure from 0.1 MPa in 0.1 MPa increments every 8 h, with continuous monitoring of the specimen’s end face for the initial appearance of water seepage. The criterion for seepage was defined as the first visible appearance of water on the specimen surface.Testing is terminated upon detecting the first instance of surface moisture penetration, and the corresponding water pressure at seepage initiation is recorded as the test result. Three replicate specimens were tested per mix, and the mean penetration pressure with standard deviation is reported.

### Quantification and statistical analysis

All statistical analyses were performed using IBM SPSS Statistics and OriginPro. Descriptive statistics are reported as mean ± standard deviation (SD) throughout the manuscript. For compressive strength, splitting tensile strength, chloride ion permeability, freeze–thaw resistance, and impermeability tests, each reported value represents the mean of three replicate specimens (*n* = 3), where n denotes the number of independently prepared and tested concrete specimens per mix group. For SEM-based porosity quantification within the interfacial transition zone (ITZ), three independent samples per concrete mix were analyzed, with 20 backscattered electron (BSE) images acquired per sample (total of 60 porosity measurements per depth point); *n* = 3 here denotes the number of independent samples. Coefficients of variation were calculated to assess measurement repeatability. Linear regression analyses were conducted to evaluate relationships between aggregate water absorption and concrete properties (chloride ion permeability, ITZ porosity, and ITZ thickness), with goodness-of-fit expressed as R^2^ values. Statistical significance was set at α = 0.05. Detailed statistical parameters—including exact *n* values, definitions of n, and dispersion measures—are provided in the figure legends (Figures 3, 4, 5, 6, 7, and 8), table notes (Tables 1, 2, 3, 4 and tables in ”raw materials and mix proportion“ section), and the results section.
